# TRIM27 interacts with Iκbα to promote the growth of human renal cancer cells through regulating the NF-κB pathway

**DOI:** 10.1186/s12885-021-08562-5

**Published:** 2021-07-20

**Authors:** Chengwu Xiao, Wei Zhang, Meimian Hua, Huan Chen, Bin Yang, Ye Wang, Qing Yang

**Affiliations:** Department of Urology, Changhai Hospital, Naval Medical University, Changhai Road No.168, Yangpu District, Shanghai, 200433 People’s Republic of China

**Keywords:** Renal cell carcinoma, TRIM27, Proliferation, Apoptosis, NF-κB, Iκbα

## Abstract

**Background:**

The tripartite motif (TRIM) family proteins exhibit oncogenic roles in various cancers. The roles of TRIM27, a member of the TRIM super family, in renal cell carcinoma (RCC) remained unexplored. In the current study, we aimed to investigate the clinical impact and roles of TRIM27 in the development of RCC.

**Methods:**

The mRNA levels of TRIM27 and Kaplan–Meier survival of RCC were analyzed from The Cancer Genome Atlas database. Real-time PCR and Western blotting were used to measure the mRNA and protein levels of TRIM27 both in vivo and in vitro. siRNA and TRIM27 were exogenously overexpressed in RCC cell lines to manipulate TRIM27 expression.

**Results:**

We discovered that TRIM27 was elevated in RCC patients, and the expression of TRIM27 was closely correlated with poor prognosis. The loss of function and gain of function results illustrated that TRIM27 promotes cell proliferation and inhibits apoptosis in RCC cell lines. Furthermore, TRIM27 expression was positively associated with NF-κB expression in patients with RCC. Blocking the activity of NF-κB attenuated the TRIM27-mediated enhancement of proliferation and inhibition of apoptosis. TRIM27 directly interacted with Iκbα, an inhibitor of NF-κB, to promote its ubiquitination, and the inhibitory effects of TRIM27 on Iκbα led to NF-κB activation.

**Conclusions:**

Our results suggest that TRIM27 exhibits an oncogenic role in RCC by regulating NF-κB signaling. TRIM27 serves as a specific prognostic indicator for RCC, and strategies targeting the suppression of TRIM27 function may shed light on future therapeutic approaches.

**Supplementary Information:**

The online version contains supplementary material available at 10.1186/s12885-021-08562-5.

## Background

In kidney cancer, renal cell carcinoma (RCC) is the leading histological type and has the highest death rate of all solid urological tumors [1]. Advances in imaging technology and an increase in physical check-ups have led to a higher incidence of RCC detection, which enables early clinical intervention [2]. Despite new approaches to RCC treatment, including new pharmacological inhibitors, RCC remains chemotherapy-resistant, and patient survival largely depends on surgical treatment at early stages [3]. Discovery of a novel therapeutic target to improve prognosis in RCC patients is an urgent clinical need. Clarification of the molecular mechanisms underlying RCC will provide evidence to improve clinical strategies.

Tripartite motif (TRIM) family proteins contribute to cancer development by mediating cell growth, metastasis, and oncogenesis [4]. TRIM27 was first identified as a regulator in oncogenic rearrangements, and contains several domains: a Really Interesting New Gene (RING) finger, two B-boxes (type I and type II), and the RBCC motif [5]. The RING finger domain, which is responsible for E3 ubiquitin activity, is directly responsible for ubiquitination [6]. As an oncogene, TRIM27 promotes cancer cell growth in various cancers, including esophageal, lung, and colorectal cancers [7, 8]. TRIM44 is another TRIM member that exhibits oncogenic roles in RCC, and higher TRIM44 levels are associated with poor prognosis [9]. However, the role of TRIM27 in RCC has not yet been investigated.

Nuclear factor kappa B (NF-κB) is an essential signaling transcription factor that regulates diverse cellular processes. NF-κB promotes proliferation, regulates cell death, stimulates migration, and mediates inflammatory processes depending on the cell type, developmental stage, and pathological state [10, 11]. The NF-κB complex is inactivate in the cytoplasm, and upon stimulation, the NF-κB dimer moves to the nucleus, where it binds to various target genes [12]. Iκbα is a well-characterized NF-κB inhibitor, which acts by removing NF-κB from DNA in the nucleus and relocating it to the cytosol [13, 14]. The activation/translocation of NF-κB requires the removal of Iκbα by ubiquitination and degradation. In lung carcinoma, NF-κB and Iκbα signaling is regulated by TRIM family members [15, 16]. Since the interaction between NF-κB/Iκbα and TRIM27 has been previously reported in other systems [17], we hypothesize that TRIM27 promotes RCC development through its effect on NF-κB/Iκbα pathways.

## Methods

### Cell culture

HK-2, 786–0, A498, ACHN, CaKi-1, and CaKi-2 cells were purchased from Shanghai Biology Institute (Shanghai, P.R. China). Cells were maintained in DMEM media (Trueline, Kaukauna, WI, USA) containing 10% FBS (Thermo Fisher Scientific, Waltham, MA, USA), L-glutamine (2 mM) and 1% penicillin-streptomycin (Solarbio; Beijing, P.R. China). Cells were placed in a 37 °C incubator with 95% air and 5% CO_2_. The NF-κB inhibitor was dissolved in DMSO (D2605, Sigma, USA).

### qRT-PCR

Total RNA was extracted using TRIzol (Invitrogen, Waltham, MA, USA). cDNA was generated using a cDNA synthesis kit (Thermo Fisher Scientific, Waltham, MA, USA). Real-time PCR was conducted with following cycling scheme: 95 °C for 10 min, 40 cycles of 95 °C for 15 s, and 60 °C for 45 s on the real-time PCR equipment (ABI-7300, ABI, USA). GAPDH was the internal control. The 2^−ΔΔCt^ method was used for gene expression analysis as mentioned in a previous report [18]. All experiments were performed using three replicates. The primer sequences were as follows: F: 5′-GAGCAAATCCAGAACCAG-3′, R 5′-TCAAACTCCCAAACAATC-3′; GAPDH: F 5′-AATCCCATCACCATCTTC-3′, R 5′-AGGCTGTTGTCATACTTC-3′; nuclear factor kappa B subunit 1 (NF-κB), F 5′-CGGGGACGGTCTGAATGC-3′, R 5′-CTGCCAATGAGATGTTGTCGTG-3′.

#### Bioinformatics

The gene expression of TRIM27 and Kaplan–Meier analysis were downloaded from TCGA dataset (http://ualcan.path.uab.edu/analysis.html) to analyze the effect of TRIM27 on overall survival of RCC patient. KEGG, REACTOME, and DUTTA analysis indicated that cell apoptosis was positively correlated with elevated TRIM27 expression in RCC from the database GSE53757. Western blotting.

Proteins were extracted in RIPA lysis buffer (JRDUN, Shanghai, P.R. China), in the presence of Protease Inhibitor Cocktail (EDTA-free; Roche, Heidelberg, Germany). The concentration was calculated by a BCA protein assay kit (Thermo Fisher Scientific, Waltham, MA, USA). A total of 25 μg of proteins were separated by 10% SDS-PAGE gels before being transferred to nitrocellulose membranes (Millipore, Billerica, MA, USA) overnight. The membranes were blocked with 5% non-fat dry milk (1 h at room temperature). Primary antibodies were incubated at 4 °C overnight, followed by incubation of secondary antibody anti-mouse IgG (1:1000; Beyotime, Shanghai, P.R. China) for 1 h at 37 °C. The bands were detected using an enhanced chemiluminescence system (Tanon, Shanghai, P.R. China). The primary antibodies that used in this study were listed as follows: TRIM27 (#15099; CST, USA), NF-kB (#8242; CST, USA), cleaved caspase 3 (Ab32042; Abcam, UK), H3 (#4499; CST, USA), GAPDH (#5174; CST, USA). IkBa (ab32518, abcam, USA). Protein expression was quantified according to the gray value after normalized to GAPDH, nuclear extract was normalized against that of histone H3.

### Knockdown and overexpression of TRIM27

Lentiviral plasmids (pLKO.1) with three siRNAs targeting human TRIM27 (NM_006510.5) and a control siRNA (siNC) were designed and generated (Major, Shanghai, People’s Republic of China). Lentiviral plasmids (pLVX-puro) with full length human TRIM27 or a mock plasmid (negative control) were generated and transiently transfected into Caki-1, Caki-2, and 786–0 cells using Lipofectamine 2000 (Thermo Fisher Scientific, Waltham, MA, USA). Cells were harvested 48 h post-transfection. The TRIM27 siRNAs were as follows: siTRIM27–1: 941–959, CCCAGTTCTCTTGCAACAT); siTRIM27–2: 1052–1070, GGGCTGAAAGAATCAGGAT; and siTRIM27–3: 1495–1513, GGATTCTGGGCAGTGTCTT.

### Cell proliferation

Cell Counting Kit-8 (CCK-8) assay kits (SAB, College Park, MD, USA) were purchased for the cell proliferation assay. Briefly, cells were transfected and seeded in 96-well plates for various time points. CCK-8 solution (1: 10) was incubated for 1 h before harvest. The optical densities were measured using a microplate reader (Pulangxin, Beijing, P.R. China) at 450 nm.

### Cell apoptosis

Caki-1, Caki-2, and 786–0 cells were collected and examined using an Annexin V-fluorescein isothiocyanate’ apoptosis kit (Beyotime, Shanghai, P.R. China) 48 h post viral infection. Cells were examined by flow cytometry (BD, San Diego, CA, USA).

### Co-immunoprecipitation (CO-IP)

Cell extracts were incubated with different antibodies in the presence of Protein A/G beads (Santa Cruz Biotechnology, Dallas, TX, USA) overnight. The beads were washed thoroughly and the elutes were examined using SDS-PAGE and Western blot.

### Ubiquitination

siNC or siTRIM27 was transfected. Cells were lysed in RIPA buffer (1% SDS) by sonication. Lysates were incubated with Protein A/G PLUS-agarose (sc-2003; Santa Cruz Biotechnology, USA) for 1 h. IgG (sc-2027; Santa Cruz Biotechnology, USA) was added to all samples and incubated overnight at 4 °C. Nuclear pellets were collected following centrifugation at 3000 rpm at 4 °C, followed by washing with Protein A/G Plus-Agarose beads four times. Proteins were loaded onto 4–20% gradient SDS-PAGE gels. Signals were detected by anti-IκBα antibody (#4812; Cell Signaling Technology, Danvers, MA, USA) and anti-ubiquitin antibody (ab7780; Abcam, UK).

### Xenograft model

All animal procedures were performed according to the Institutional Animal Care and Use Committee. Experiments were approved by the independent ethics committee of the Changhai Hospital, Naval Medical University, Shanghai. siNC- or siTRIM27-transfected Caki-2 cells (*n* = 2 × 10^6^), and oeNC or oeTRIM27-transfected Caki-1 cells (*n* = 2 × 10^6^) were injected into the right flank of nude mice (4–6 weeks old, *n* = 6 in each group; Shanghai Laboratory Animal Company, Shanghai, PR China). Mice were sacrificed by cervical dislocation after 6 weeks. Mouse tissues were collected in 4% formalin.

### TUNEL

TUNEL assays were performed on sections using an ApopTag kit (11,684,817,910; Roche, Switzerland) principally according to the supplier’s instructions. Three independent biological replicates were required for each sample.

### Histopathology

Tissues were fixed in 10% formalin for 48 h before embedding in paraffin. Tissues were processed into slices using a microtome (Leike, China). A series of xylene and alcohol incubations were performed. Tissues were observed by hematoxylin and eosin staining.

### Immunohistochemistry

Tissues were embedded in paraffin. Slices (5 μm) were treated in H_2_O_2_ (3%) dissolved in methanol and 5% normal horse serum before staining. Sections were incubated with the following primary antibodies overnight at 4 °C: anti-TRIM27 antibody (ab137638; Abcam, UK) and anti-NF-KB antibody (ab16502; Abcam, UK). The sections were then incubated with secondary goat anti-rabbit IgG (1:100; ab64256; Abcam) for 30 min at room temperature. The sections were visualized with diaminobenzidine and hematoxylin. Cells with > 25% positive staining were defined as having “high expression.”

### Statistical analysis

Analysis was performed by GraphPad Prism Version 7.0 (La Jolla, CA, USA). Data are shown as mean ± SD. One-way ANOVA was performed, and *P*-values < 0.05 indicated statistical significance.

## Results

### Increased expression of TRIM27 in RCC correlates with poor prognosis

**Fig. 1:**
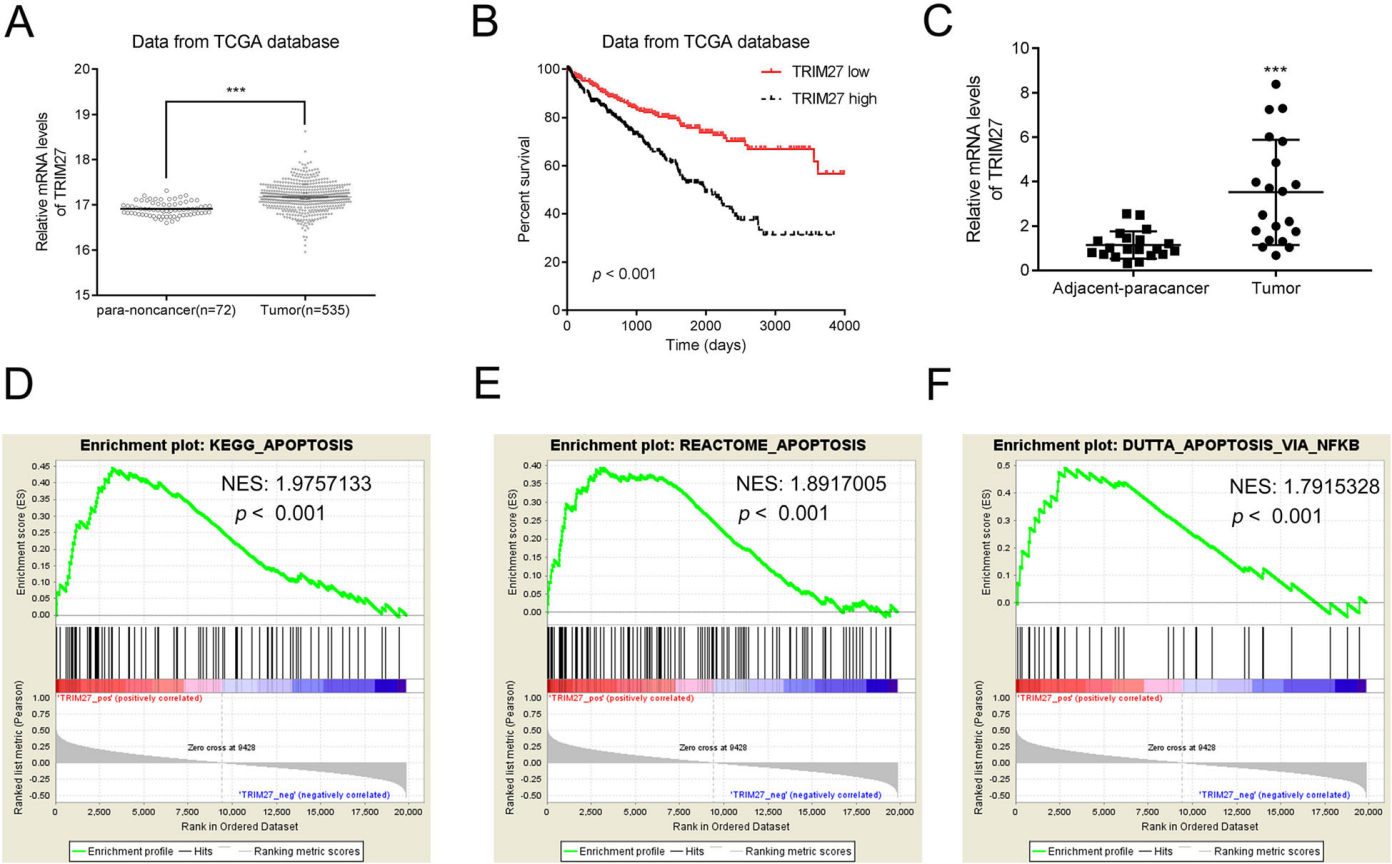
TRIM27 is upregulated and correlated with poor prognosis in human renal cancer. **A**. The mRNA level of TRIM27 was upregulated in renal cancer. Data were collected from the TCGA database. ***p < 0.001 vs pare-non-cancer tissues **B**. Prognosis of RCC (KIRC) patients with high or low expression of TRIM27 derived from the TCGA database. **C**. The relative mRNA level of TRIM27 was upregulated in human renal cancer tissues. *n* = 20, ***p < 0.001 vs pare-non-cancer tissues. **D**–**F**. KEGG, REACTOME, and DUTTA analysis indicated that cell apoptosis was positively correlated with elevated TRIM27 expression in RCC from the database GSE53757. NES: Normalized enrichment score

To define TRIM27 expression in RCC, we first compared the TRIM27 mRNA from RCC tissues (*n* = 535) and normal renal tissues (*n* = 72) from The Cancer Genome Atlas (TCGA). Our results showed that TRIM27 mRNA was significantly elevated in RCC tissues (Fig. 1A). Consistent with RCC patient data, TRIM27 was selectively upregulated in human RCC cell lines (Fig. S1A). Western blots showed that TRIM27 was increased in 786–0 and Caki-1 cells at translational levels (Fig. S1B). We conducted a chi-square test to investigate any correlation between TRIM27 and RCC clinical characteristics. High levels TRIM27 were associated with several clinical characteristics, including tumor size, T stage, Fuhrman grade, and metastasis (Table 1).

**Table 1:** Correlation between TRIM27 expression and clinicopathologic features in patients with renal cancer

Parameters	Group	N	TRIM27 expression		*P* value
			Low	High	
Age(years)	< 60	46	29	17	ns
	≥ 60	55	28	27	
Gender	Male	53	29	24	ns
	Female	48	28	20	
Tumor Side	Left	49	27	22	ns
	Right	52	30	22	
Tumor Size (cm)	≤4	59	38	21	*
	>4	42	19	23	
T stage	T1–2	72	46	26	*
	T3–4	29	11	18	
Fuhrman	I-II	76	47	29	*
	III-IV	25	10	15	
Metastasis	Negative	74	46	28	*
	Positive	27	11	16	

* *p* < 0.05, *p* value from Chi-square test

Kaplan–Meier analysis of TCGA datasets demonstrated that the survival time of patients with high mRNA levels of TRIM27 was significantly shorter than that of patients with lower levels (Fig. 1B). These results suggest that up-regulation of TRIM27 expression in RCC links to a shorter survival duration. To confirm the TCGA data analysis, we measured TRIM27 mRNA levels in 20 RCC patients, and adjacent normal tissues from the same patient were also collected as the negative control. TRIM27 was notably elevated in tumor tissues (Fig. 1C). To identify pathways affected by TRIM27, we performed Gene Set Enrichment Analysis (GSEA) using a TCGA dataset. Our data illustrated that TRIM27 expression was positively associated with apoptosis pathways (Fig. 1D–F). Since disrupted apoptosis pathways play essential roles in neoplastic progression, TRIM27 might be a future biomarker to predict RCC prognosis. To gain a better understanding of the clinical significance of TRIM27, we studied the biological actions of TRIM27 using RCC cell lines, and by altering TRIM27 expression in vivo*.*

### Suppression of TRIM27 inhibits cell proliferation and promotes apoptosis

**Fig. 2:**
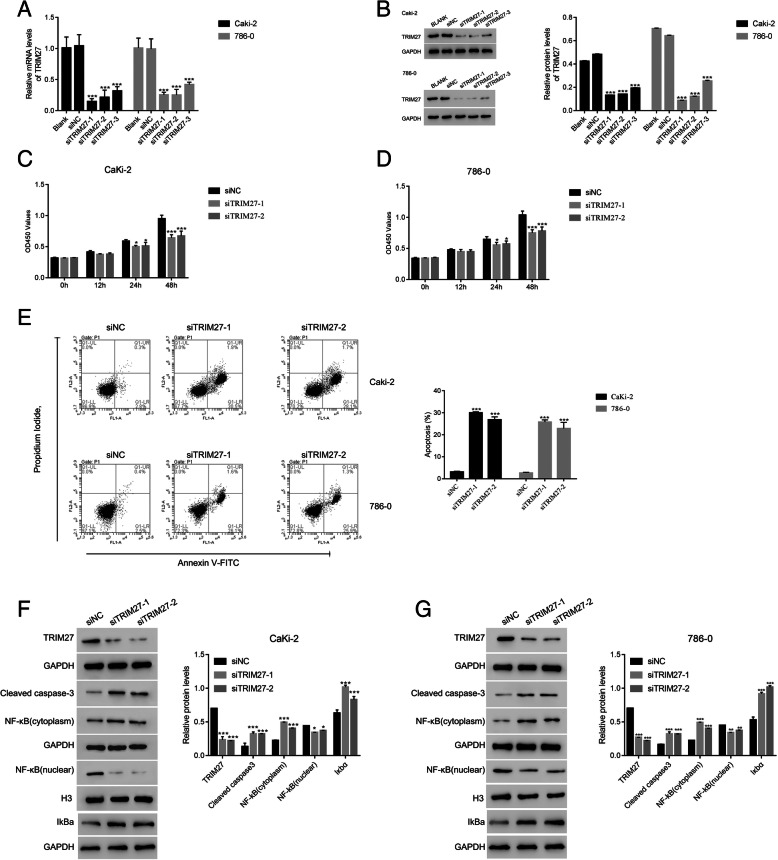
TRIM27 silencing suppressed the proliferation and promoted apoptosis of human renal cells. **A** & **B**. TRIM27 siRNAs deeply suppressed the relative mRNA and protein content of TRIM27 in human Caki-2 and 786–0 cells, respectively. ***p < 0.001 vs siNC. The mRNA levels of TRIM27 was used 2^−ΔΔCt^ method and normalized to that of GAPDH. **C** & **D**. siTRIM27–1 and siTRIM27–2 significantly suppressed the proliferation of Caki-2 and 786–0 cells. *p < 0.05 vs siNC, ***p < 0.001 vs siNC. **E**. Apoptosis of Caki-2 and 786–0 cells was significantly increased following transfecting with siTRIM27–1 and siTRIM27–2 at 48 h, respectively. ***p < 0.001 vs siNC. **F** & **G**. Western blot was used to examine the protein contents of TRIM27, cleaved caspase-3, NF-κB (cytoplasm), NF-κB (nuclear) and IkBa in Caki-2 cells (**F**) and 786–0 cells (**G**) transfected with siNC, siTRIM27–1, or siTRIM27–2 at 48 h respectively. *p < 0.05 vs siNC, **p < 0.01 vs siNC, ***p < 0.001 vs siNC

We designed three small interference RNAs (siRNAs) targeting TRIM27 and examined the effects on Caki-2 and 786–0 cells with a relatively higher level of TRIM27. All three siRNAs effectively inhibited the mRNA and protein expression of TRIM27 (Fig. 2A, B). siRNA-1 and siRNA-2 exhibited a higher knockdown efficiency than siRNA-3, and these were selected for subsequent assays. We used CCK-8 to determine the effects of TRIM27 on cell proliferation and found that cell growth was dramatically suppressed in TRIM27-suppressed groups in both Caki-2 (Fig. 2C) and 786–0 cells (Fig. 2D).

We next used Annexin V-FITC/PI staining to evaluate the effects of TRIM27 on apoptosisand found that suppression of TRIM27 in RCCs promoted cell apoptosis (Fig. 2E). We then measured apoptotic promoting protein (caspase-3) and found an elevation in caspase-3 in TRIM27 suppressed Caki-2 and 786–0 cells (Fig. 2F, G). The role of NF-κB translocation to the nucleus in inhibition of apoptosis is well defined [10]; therefore, we examined the subcellular location of NF-κB as another indicator of apoptosis. Our results demonstrated that NF-κB was less active in the presence of TRIM27 siRNAs (Fig. 2F, G), confirming that apoptosis was increased. Moreover, our results suggested that knockdown of TRIM27 promoted the expression of IkBa in Caki-2 and 786–0 cells respectively.

### OeTRIM27 rescue the function of TRIM27 in siTRIM27-transfected cells

**Fig. 3:**
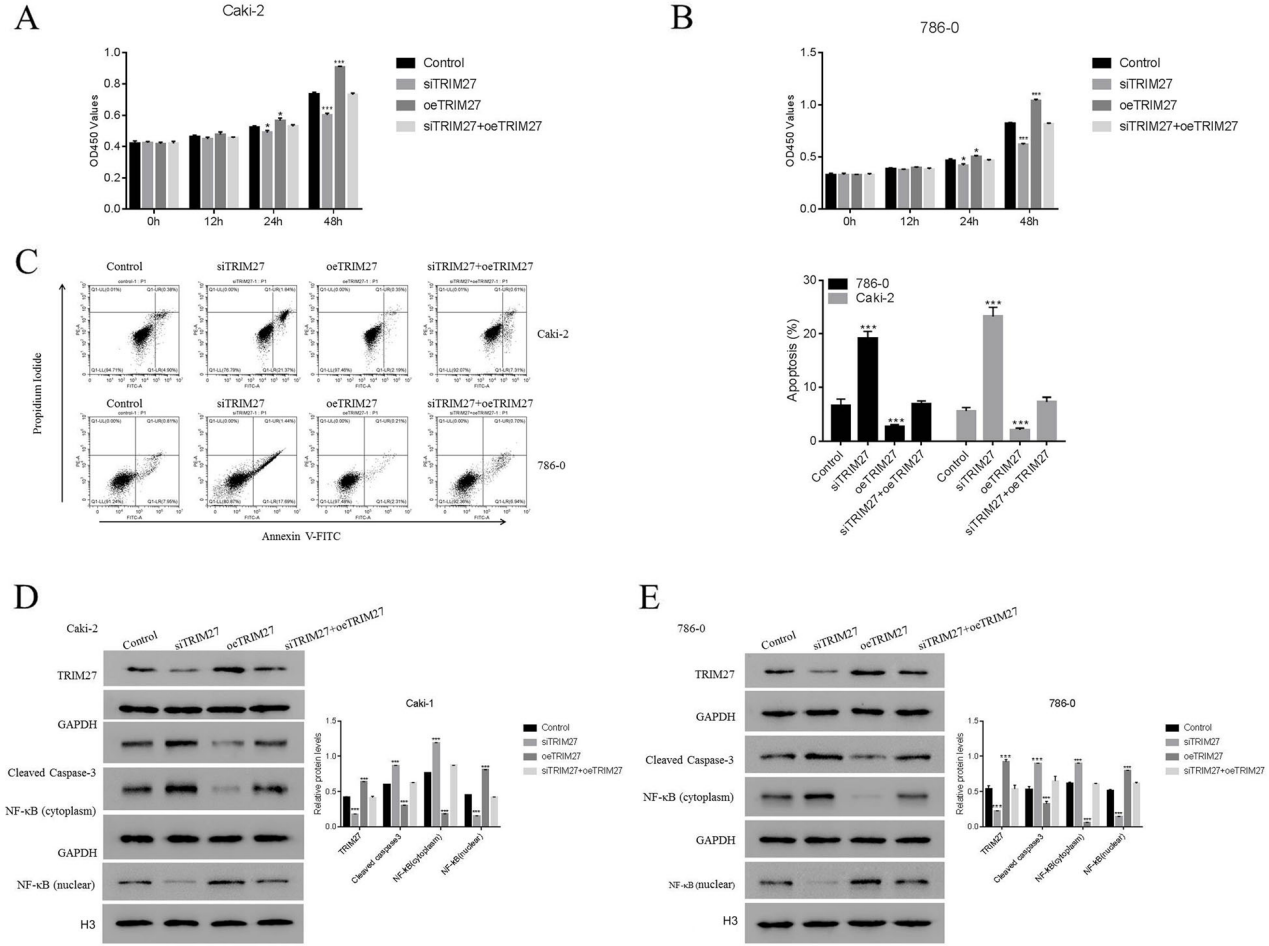
oeTRIM27 rescues the function of TRIM27 in siTRIM27-transfected cells. **A** and **B**. The proliferations of Caki-2 and 786–0 cells transfected with siTRIM27, oeTRIM27, or siTRIM27 + oeTRIM27 at 0, 12, 24, and 48 h respectively. *p < 0.05 vs Control, ***p < 0.001 vs Control. **C** and **D**. Flow cytometric evaluation of the apoptosis of Caki-2 and 786–0 cells transfected with siTRIM27, oeTRIM27, or siTRIM27 + oeTRIM27 at 48 h respectively, ***p < 0.001 vs Control. **D** and **E**. Western blot was used to examine the relative proteins of TRIM27, cleaved caspase-3, NF-κB (cytoplasm), and NF-κB (nuclear) in Caki-2 and 786–0 cells transfected with siTRIM27, oeTRIM27, or siTRIM27 + oeTRIM27 at 48 h respectively. ***p < 0.001 vs Control

To further verify our results, oeTRIM27 was used to rescue the function of TRIM27 in siTRIM27-transfected cells. As shown in Fig. 3A and B, TRIM27 overexpression significantly increased the proliferation rate of Caki-2 and 786–0 cells compared to control cells. Importantly, the proliferation of siTRIM27 cells were also increased after transfection with oeTRIM27, but showed no significant difference compared to control cells. Moreover, transfecting siTRIM27-transfected Caki-2 and 786–0 cells with oeTRIM27 led to an increase in apoptosis (Fig. 3C). Furthermore, the protein level of TRIM27 was significantly recovered by oeTRIM27 in siTRIM27-transfeced cells. More importantly, the protein levels of cleaved caspase-3, NF-κB (cytoplasm), and NF-κB (nuclear) in siTRIM27-transfected cells were similar following transfection with oeTRIM27 (Fig. 3D, E). Taken together, these results suggest that the function of TRIM27 is rescued by oeTRIM27 in siTRIM27-transfected cells.

### Suppression of TRIM27 reduces tumor growth in vivo

**Fig. 4:**
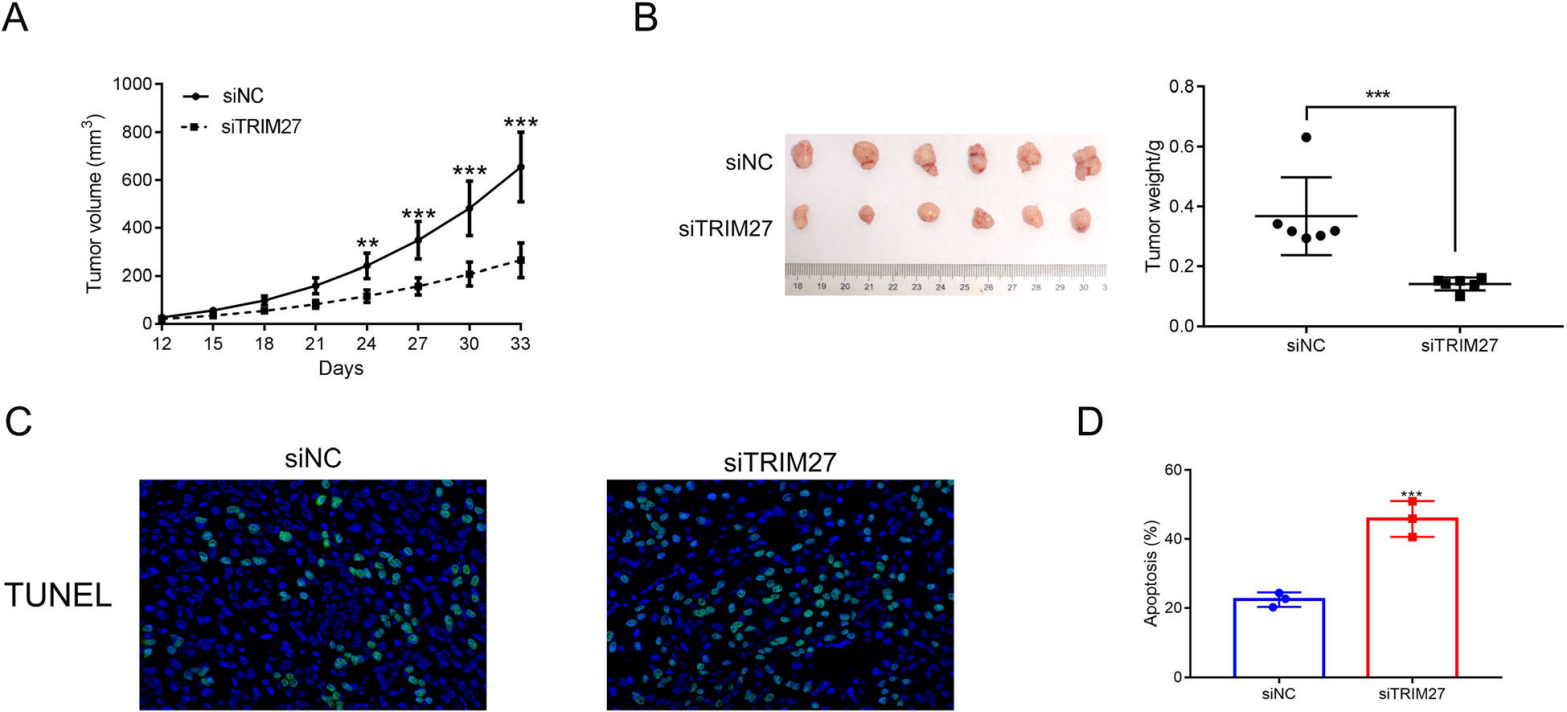
TRIM27 silencing reduced the tumorigenicity of human Caki-2 cells in vivo. **A** & **B**. Knockdown of TRIM27 in Caki-2 cells deeply suppressed the tumor volume and weight in vivo. **p < 0.01 vs siNC, ***p < 0.001 vs siTRIM27. **C**. TUNEL staining assays were used to determine tumor cell apoptosis. **D**. TUNEL quantification of siNC and siTRIM27 tumors, ***p < 0.001 vs siNC

Next, we examined whether TRIM27 siRNAs reduce tumor development in vivo. Caki-2 cells transfected with siNC or siTRIM27 were injected into nude mice and the tumor tissues were measured on day 33. TRIM27-suppressed tissues exhibited a slower growth rate than the controls (Fig. 4A), while the tumor weight was reduced in the siTRIM27 group (Fig. 4B). We next performed a TUNEL assay to establish whether the decreased tumor growth was due to apoptosis. Our results illustrated that apoptosis was increased in the siTRIM27 group compared to the control group. These findings suggest that suppression of TRIM27 prevents tumor growth in vivo, partially due to increased tumor cell apoptosis (Fig. 4C-D).

**Fig. 5:**
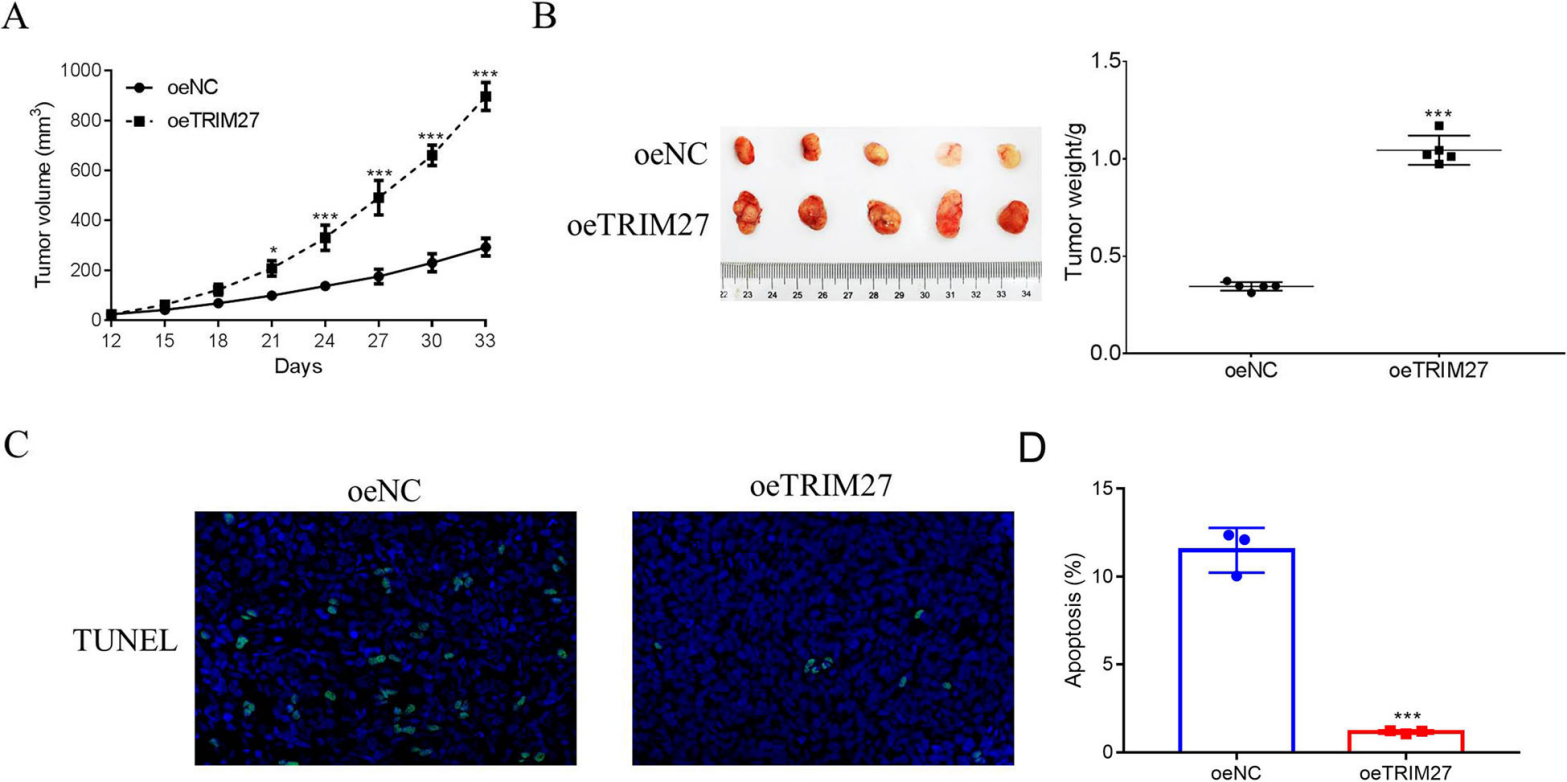
TRIM27 overexpression promoted the tumorigenicity of human Caki-1 cells in vivo. **A** & **B**. Overexpression of TRIM27 in Caki-2 cells significantly increased the tumor volume and weight in vivo. *p < 0.05 vs oeNC, ***p < 0.001 vs oeNC. **C**. TUNEL staining assays were used to determine tumor cell apoptosis. **D**. TUNEL quantification of oeNC and oeTRIM27 tumors, ***p < 0.001 vs oeNC

To further determine the function of TRIM27 in vivo*,* the oeNC or oeTRIM27 transfected Caki-1 cells were used to construct a xenograft model. As showed in Fig. 5A and B, both the oeNC and oeTRIM27 transfected cells were able to develop into tumors. Moreover, both the tumor volume and weight were increased in oeTRIM27 tumors. TUNEL staining was performed to examine apoptosis in oeNC and oeTRIM27 tumors, and we found that overexpression of TRIM27 significantly suppressed the apoptosis of Caki-1 cells in vivo (Fig. 5C-D). Taken together, our data illustrate that TRIM27 promotes RCC tumorigenesis and exhibits anti-apoptotic effects in vivo*.*

### NF-κB signaling is essential for overexpressing TRIM27 signaling

**Fig. 6:**
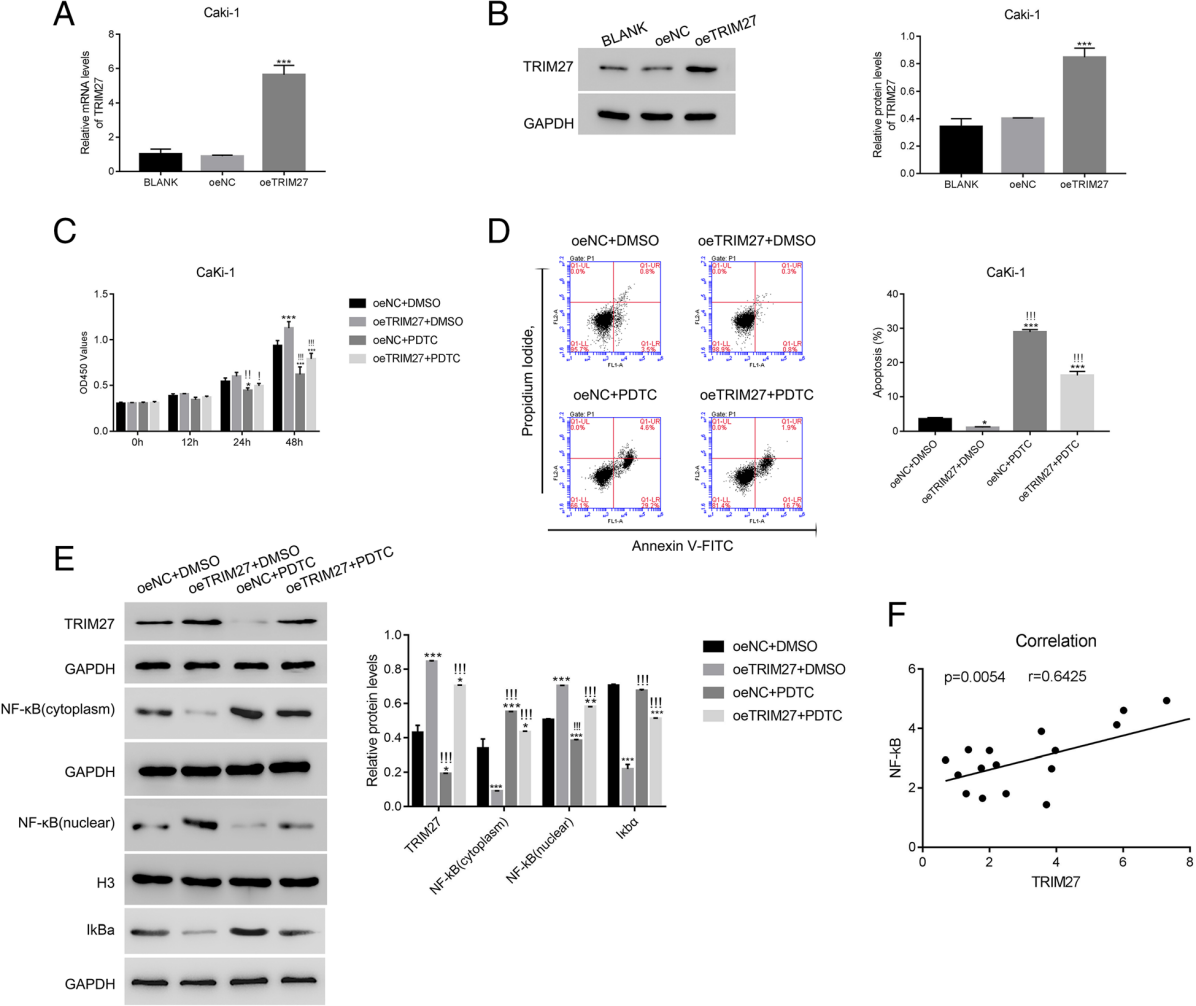
The NF-κB inhibitor PDTC suppressed the function of oeTRIM27 in Caki-1 cells. **A** & **B**. oeTRIM27 significantly upregulated the relative mRNA and protein levels of TRIM27 in Caki-1 cells. ***p < 0.001 vs oeNC. The mRNA levels of TRIM27 was used 2^−ΔΔCt^ method and normalized to that of GAPDH. **C**. The proliferation of oeTRIM27 transfected cells was deeply downregulated in the presence of the inhibitor PDTC. *p < 0.05 vs oeNC + DMSO, ***p < 0.001 vs oeNC + DMSO;!*p* < 0.05 vs oeTRIM27 + DMSO,!!*p* < 0.01 vs oeTRIM27 + DMSO,!!!*p* < 0.001 vs oeTRIM27 + DMSO. **D**. The inhibitor PDTC significantly reduced the apoptosis of oeTRIM27 transfected cells. *p < 0.05 vs oeNC + DMSO, ***p < 0.001 vs oeNC + DMSO;!!!*p* < 0.001 vs oeTRIM27 + DMSO. **E**. The nuclear translocation of NF-κB and IkBa in oeNC or oeTRIM27 transfected cells were deeply suppressed by the inhibitor PDTC. ***p < 0.001 vs oeNC + DMSO;!!!*p* < 0.001 vs oeTRIM27 + DMSO. **F**. Correlation analysis between TRIM27 and NF-κB in human renal cancer tissues (*n* = 20)

Virus-mediated TRIM27 overexpression (oeTRIM27) significantly enhanced TRIM27 expression in Caki-1 cells, both at the transcriptional (Fig. 6A) and translational (Fig. 6B) levels., oeTRIM27 promoted cell proliferation at 48 h (Fig. 6C) and inhibited apoptosis (Fig. 6D). Since we know that NF-κB activation is altered by TRIM27 (Fig. 2F), we sought to determine whether NF-κB mediates the actions of TRIM27. To address this question, we induced chemical inhibition of NF-κB signaling using ammonium pyrrolidinedithiocarbamate (PDTC); our results showed that cell proliferation was blocked at 24 h and 48 h as a result, and the oeTRIM27 group did not differ from the control group in the presence of PDTC (Fig. 6C). These findings suggest that NF-κB is involved in TRIM27 signaling. The application of PDTC induced apoptosis, which was attenuated by oeTRIM27 (Fig. 6D). oeTRIM27 activated NF-κB, which was suppressed by PDTC administration (Fig. 6E). Importantly, the inhibitor PDTC also suppressed the protein level of TRIM27 in oeTRIM27 transfected cells, while promoted the expression of IkBa.

We also analyzed the correlation between TRIM27 and NF-κB using human renal cancer tissues. We found there was a linear correlation between levels of TRIM27 and NF-κB (Fig. 6F). These data suggest that NF-κB is a potential upstream regulator of TRIM27 in RCC. We induced siRNA-mediated knockdown of NF-κB to further explore this connection, and qRT-PCR and Western blot were used to examine the relative mRNA and protein levels of NF-κB and TRIM27 in Caki-1 cells. As shown in Fig. S2A, B, both the relative mRNA and protein levels of NF-κB and TRIM27 were suppressed by NF-κB siRNAs.

### TRIM27 interacts with Iκbα and is positively associated with Iκbα ubiquitination

**Fig. 7:**
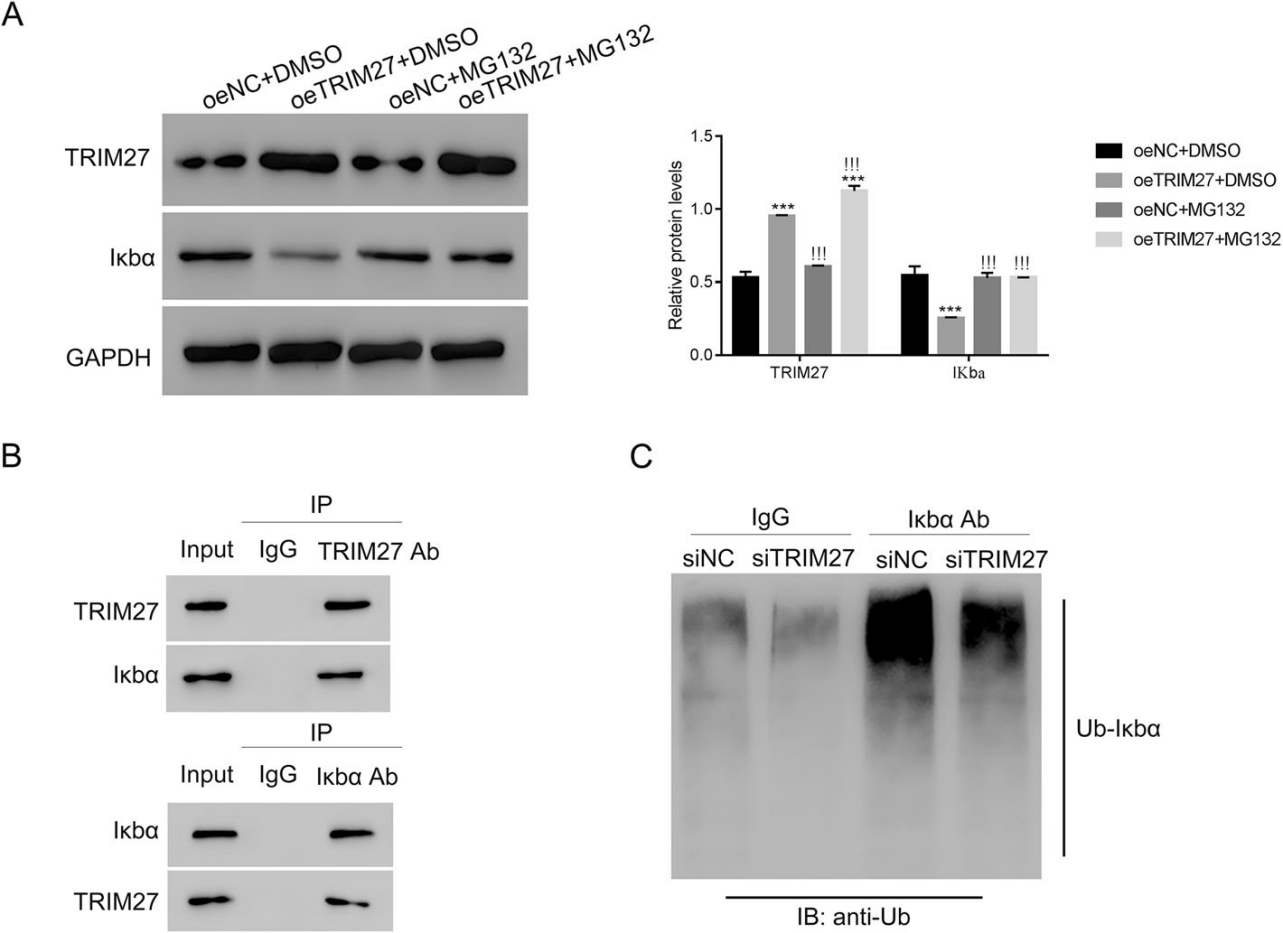
TRIM27 interacted with Iκbα and was positively associated with its ubiquitination in human Caki-2 cells. **A**. Western blot was used to examine the protein content of TRIM27 and Iκbα in oeNC or oeTRIM27 with or without the MG132 treatment. ***p < 0.001 vs oeNC + DMSO;!!!*p* < 0.001 vs oeTRIM27 + DMSO. **B**. TRIM27 interacted with Iκbα in human Caki-2 cells. **C**. TRIM27 silencing suppressed the ubiquitination of Iκbα in human Caki-2 cells

Iκbα has been identified as the major regulator of the NF-κB pathway [19], and dissociation of NF-κB from Iκbα is a critical step in translocation to the nucleus. In the current study, we found that oeTRIM27 significantly decreased Iκbα levels, and that this change was blunted in the presence of MG132, an inducer of apoptosis in cancer cells (Fig. 7A). We used co-immunoprecipitation (Co-IP) to examine whether TRIM27 interacts with Iκbα (Fig. 7B), and our results showed a direct interaction. Ubiquitination is essential for the stability of Iκbα, as well as its location in the nucleus. Moreover, increasing Iκbα ubiquitination improves NF-κB activation [20]. We demonstrated that Iκbα ubiquitination was remarkably attenuated by siTRIM27 (Fig. 7C), suggesting that TRIM27 promotes Iκbα ubiquitination in RCC.

## Discussion

RCC is the most common type of kidney cancer and remains the deadliest of all urologic cancers. Although numerous approaches have been developed to improve understanding of the molecular signaling pathways and underling mechanisms, the use of molecular-targeted therapy in RCC is limited due to the lack of therapeutic candidates. In the current study, we identified TRIM27 as a novel molecular target of RCC and examined the potential uses of TRIM27 as a candidate target for effective molecular-targeted therapeutics for RCC. Present findings reveal a novel underling mechanism between TRIM27 and Iκbα in oncogenesis, which we believe will assist with future therapeutic approaches toward RCC treatment.

TRIM superfamily proteins have been proven to regulate diverse cellular processes in cancer biology. As a diverse family, members of the TRIM superfamily exhibit a variety of roles in oncogenesis. TRIM44 and TRIM59, identified as oncogenes, are enriched in cancers and involved ininvasion stimulus, which predicts poor prognosis [9, 21]. In sharp contrast, TRIM2 and TRIM13, are dramatically decreased in RCC and act as antioncogenes by inhibiting migration, cell proliferation, and metastasis [22, 23]. The results of our study provide novel evidence that TRIM27 is another example of a TRIM superfamily member that has an oncogenic role in cancer. Similar to the other oncogenic TRIM proteins, TRIM27 overexpression in RCC significantly inhibited apoptosis and predicted poor survival. Our results confirmed the hypothesis that TRIM proteins possess the potential to serve as new therapeutic targets for cancer therapy.

Although the majority of TRIM superfamily members share a TRIM motif, this protein domain is seen in isolation or in combination with a variety of C-terminal domains. For diverse functions, the TRIM superfamily encodes proteins that are divided into 11 distinct subfamilies. The distinctly different actions of TRIM27 as compared to other TRIM superfamily members are likely due to the diversity of protein structures. TRIM27 belongs to the RET/PTC (Rearranged in Transformation/Papillary Thyroid Carcinomas) fusion protein subfamily that is involved in oncogenic actions, and the RET proto-oncogene domain of this subfamily is considered essential for its oncogenic potential [24, 25]. Moreover, two other TRIM proteins (TRIM33 and TRIM24) have been reported as oncogene or oncogenic transcriptional activators in B-cell leukemias and prostate cancer, respectively [26, 27]. Thus, the results of our study are consistent with those of previously published findings, in that they suggest that RET/PTC subfamily proteins from the TRIM superfamily are likely to have oncogenic roles in cancer.

TRIM27 expression is a known oncogene and disease progression in various cancers, such as lung, ovarian, colon, and esophageal cancer [5, 7, 28, 29]. Indeed, TRIM27 has been shown to promote tumor invasion and growth in colon cancer through its promotion of the epithelial-mesenchymal transition and activation of the AKT pathway [5]. Similarly, the TRIM27 transcript level was found to be correlated with ovarian cancer metastasis, and it has been shown that TRIM27 knockdown induces cell cycle arrest by activating the p38 pathway [29]. Unlike other cancer types, the exact molecular mechanisms underlying the oncogenic action of TRIM proteins in RCC remain unclear. However, similar to the molecular biological features of TRIM27 in esophageal and colon cancer [5, 7], we identified similar biological functions of TRIM27 in that it is significantly elevated in RCC tissues, accelerates the pathogenesis of RCC, and predicts RCC progression and poorer survival. In addition to its previously identified roles in other cancers, the present study indicates that TRIM27 has oncogenic roles in RCC and could serve as a predictive marker and therapeutic target for a variety types of cancers, including, but not limited to RCC.

Many downstream signaling pathways of TRIM27 have been identified in cancer, including PTEN/AKT, p38, and STAT3 [7, 8, 29]. Constitutive activation of NF-κB signaling mediates tumorigenesis and poor prognosis in RCC. Our study demonstrated that TRIM27 positively mediates NF-κB signaling pathway by degradation of the NF-κB inhibitor Iκbα. In contrast to our findings, two previous studies found that TRIM13, another TRIM protein, inactivated NF-κB in both RCC and non-small-cell lung carcinoma [22, 30]. The opposing regulation of these TRIM family members in NF-κB activity again indicates that different subfamily proteins of the TRIM superfamily may utilize different mechanisms and pathways in cancer development. It is important to note that the previous study of TRIM13 in RCC only examined one cell line (786–0), while the present study examined more than three cell lines, all of which showed consistent results. The molecular interactions between TRIM27 and Iκbα are also supported by a study of TRIM8 that found that NF-κB signaling was enhanced by TRIM8 through polyubiquitination of serine/threonine kinases [31]. Whether other proinflammatory cytokines are involved in TRIM27 and Iκbα interactions is not clear at this time; nevertheless, the present study provides further evidence that TRIM superfamily proteins could play either positive or negative roles in NF-κB-induced cancer immunity. Further investigation is required to clarify the actions of TRIM27 through other pathways in RCC.

To the best of our knowledge, this is the first study to demonstrate that TRIM27 is increased in human patients with RCC and may serve as an effective novel prognosis marker for RCC.

## Supplementary Information

**Additional file 1 Fig. S1:** TRIM27 was upregulated in human renal cancer cells. **A**. The relative mRNA levels of TRIM27 were much higher in human renal 786–0 and Caki-2 cells than that of HK-2 cells. ** *p* < 0.01 vs HK-2, *** *p* < 0.001 vs HK-2. **B**. Western blot was performed to examine the protein contents of TRIM27 in HK-2, 786–0, A498, ACHN, CaKi-1 and CaKi-2 cells respectively. *** *p* < 0.001 vs HK-2. **Fig. S2**: siNF-κB inhibited the expression of TRIM27 in Caki-1 cells. **A** and **B**. The relative mRNA and protein levels of NF-κB and TRIM27 in Caki-1 cells that transfecting with siNC, siNF-κB-1, siNF-κB-2 and siNF-κB-3 respectively. *** *p* < 0.001 vs siNC.

## Data Availability

The datasets used and/or analyzed during the current study are available from the corresponding author on reasonable request.

## Abbreviations

TRIM27: Tripartite motif 27
RCC: renal cell carcinoma
RING: Really Interesting New Gene
NF-κB: Nuclear factor kappa B
GSEA: Genome Set Enrichment Analysis
CCK-8: Cell Counting Kit-8
CO-IP: Co-Immunoprecipitation
TCGA: The Cancer Genome Atlas

## References

[1] Schmidt AL, Siefker-Radtke A, McConkey D, McGregor B (2020). Renal cell and urothelial carcinoma: biomarkers for new treatments. Am Soc Clin Oncol Educ Book.

[2] Sankineni S, Brown A, Cieciera M, Choyke PL, Turkbey B (2016). Imaging of renal cell carcinoma. Urol Oncol.

[3] Szymanski L, Helbrecht I, Fiedorowicz M, Matak D, Bartnik E, Golik P, Szczylik C, Czarnecka AM (2019). Cancer stem cells in renal carcinoma. Postepy Biochem.

[4] Micale L, Chaignat E, Fusco C, Reymond A, Merla G (2012). The tripartite motif: structure and function. Adv Exp Med Biol.

[5] Zhang Y, Feng Y, Ji D, Wang Q, Qian W, Wang S, Zhang Z, Ji B, Zhang C, Sun Y, Fu Z (2018). TRIM27 functions as an oncogene by activating epithelial-mesenchymal transition and p-AKT in colorectal cancer. Int J Oncol.

[6] van Gent M, Sparrer KMJ, Gack MU (2018). TRIM proteins and their roles in antiviral host defenses. Annu Rev Virol.

[7] Ma L, Yao N, Chen P, Zhuang Z (2019). TRIM27 promotes the development of esophagus cancer via regulating PTEN/AKT signaling pathway. Cancer Cell Int.

[8] Zhang HX, Xu ZS, Lin H, Li M, Xia T, Cui K, Wang SY, Li Y, Shu HB, Wang YY (2018). TRIM27 mediates STAT3 activation at retromer-positive structures to promote colitis and colitis-associated carcinogenesis. Nat Commun.

[9] Yamada Y, Kimura N, Takayama KI, Sato Y, Suzuki T, Azuma K, Fujimura T, Ikeda K, Kume H, Inoue S (2020). TRIM44 promotes cell proliferation and migration by inhibiting FRK in renal cell carcinoma. Cancer Sci.

[10] Albensi BC (2019). What is nuclear factor kappa B (NF-kappaB) doing in and to the mitochondrion?. Front Cell Dev Biol.

[11] Qin ZH, Tao LY, Chen X (2007). Dual roles of NF-kappaB in cell survival and implications of NF-kappaB inhibitors in neuroprotective therapy. Acta Pharmacol Sin.

[12] Hayden MS, Ghosh S (2004). Signaling to NF-kappaB. Genes Dev.

[13] Schmitz ML, Shaban MS, Albert BV, Gokcen A, Kracht M. The Crosstalk of Endoplasmic Reticulum (ER) Stress Pathways with NF-kappaB: Complex Mechanisms Relevant for Cancer, Inflammation and Infection. Biomedicines. 2018;6(2):58. 10.3390/biomedicines6020058 PMC6027367 29772680

[14] Hoesel B, Schmid JA (2013). The complexity of NF-kappaB signaling in inflammation and cancer. Mol Cancer.

[15] Xu L, Wu Q, Zhou X, Wu Q, Fang M (2019). TRIM13 inhibited cell proliferation and induced cell apoptosis by regulating NF-kappaB pathway in non-small-cell lung carcinoma cells. Gene.

[16] Zhang Y, Du H, Li Y, Yuan Y, Chen B, Sun S (2020). Elevated TRIM23 expression predicts cisplatin resistance in lung adenocarcinoma. Cancer Sci.

[17] Kawai T, Akira S (2011). Regulation of innate immune signalling pathways by the tripartite motif (TRIM) family proteins. EMBO Mol Med.

[18] Livak KJ, Schmittgen TD (2001). Analysis of relative gene expression data using real-time quantitative PCR and the 2(−Delta Delta C(T)) method. Methods.

[19] Wan F, Lenardo MJ (2010). The nuclear signaling of NF-kappaB: current knowledge, new insights, and future perspectives. Cell Res.

[20] Chen H, Xu Z, Li X, Yang Y, Li B, Li Y, Xia K, Wang J, Li S, Wang M (2018). alpha-catenin SUMOylation increases IkappaBalpha stability and inhibits breast cancer progression. Oncogenesis.

[21] Hu SH, Zhao MJ, Wang WX, Xu CW, Wang GD (2017). TRIM59 is a key regulator of growth and migration inrenal cell carcinoma. Cell Mol Biol (Noisy-le-grand).

[22] Li H, Qu L, Zhou R, Wu Y, Zhou S, Zhang Y, et al. TRIM13 inhibits cell migration and invasion in clear-cell renal cell carcinoma. Nutr Cancer. 2020;72(7):1115–24. 10.1080/01635581.2019.1675721 31762344

[23] Xiao W, Wang X, Wang T, Xing J (2018). TRIM2 downregulation in clear cell renal cell carcinoma affects cell proliferation, migration, and invasion and predicts poor patients' survival. Cancer Manag Res.

[24] Hatakeyama S (2017). TRIM family proteins: roles in autophagy, immunity, and carcinogenesis. Trends Biochem Sci.

[25] Prescott JD, Zeiger MA (2015). The RET oncogene in papillary thyroid carcinoma. Cancer.

[26] Wang E, Kawaoka S, Roe JS, Shi J, Hohmann AF, Xu Y, Bhagwat AS, Suzuki Y, Kinney JB, Vakoc CR (2015). The transcriptional cofactor TRIM33 prevents apoptosis in B lymphoblastic leukemia by deactivating a single enhancer. eLife.

[27] Tsai WW, Wang Z, Yiu TT, Akdemir KC, Xia W, Winter S, Tsai CY, Shi X, Schwarzer D, Plunkett W, Aronow B, Gozani O, Fischle W, Hung MC, Patel DJ, Barton MC (2010). TRIM24 links a non-canonical histone signature to breast cancer. Nature.

[28] Zoumpoulidou G, Broceno C, Li H, Bird D, Thomas G, Mittnacht S (2012). Role of the tripartite motif protein 27 in cancer development. J Natl Cancer Inst.

[29] Zhu W, Ma L, Yang B, Zheng Z, Chai R, Liu T, Liu Z, Song T, Li F, Li G (2016). Flavone inhibits migration through DLC1/RhoA pathway by decreasing ROS generation in breast cancer cells. In Vitro Cell Dev Biol Anim.

[30] Xu L, Wu Q, Zhou X, Fang M (2019). TRIM13 inhibited cell proliferation and induced cell apoptosis by regulating NF-kappaB pathway in non-small-cell lung carcinoma cells. Gene.

[31] Li Q, Yan J, Mao AP, Li C, Ran Y, Shu HB, Wang YY (2011). Tripartite motif 8 (TRIM8) modulates TNFalpha- and IL-1beta-triggered NF-kappaB activation by targeting TAK1 for K63-linked polyubiquitination. Proc Natl Acad Sci U S A.

