# Supplementary material for: TRIM27 interacts with Iκbα to promote the growth of human renal cancer cells through regulating the NF-κB pathway
Source: BMC Cancer. 2021 Jul 20;21:841. doi: 10.1186/s12885-021-08562-5 (PMC8293539; doi:10.1186/s12885-021-08562-5)
Supplement: Supplementary file 1 — Additional file 1 Fig. S1: TRIM27 was upregulated in human renal cancer cells. A. The relative mRNA levels of TRIM27 were much higher in human renal 786–0 and Caki-2 cells than that of HK-2 cells. ** p < 0.01 vs HK-2, *** p < 0.001 vs HK-2. B. Western blot was performed to examine the protein contents of TRIM27 in HK-2, 786–0, A498, ACHN, CaKi-1 and CaKi-2 cells respectively. *** p < 0.001 vs HK-2. Fig. S2: siNF-κB inhibited the expression of TRIM27 in Caki-1 cells. A and B. The relative mRNA and protein levels of NF-κB and TRIM27 in Caki-1 cells that transfecting with siNC, siNF-κB-1, siNF-κB-2 and siNF-κB-3 respectively. *** p < 0.001 vs siNC. [file 12885_2021_8562_MOESM1_ESM.docx]

Title page

Title: TRIM27 interacts with Iκbα to promote the growth of human renal cancer cells through regulating the NF-κB pathway

Chengwu Xiao, Wei Zhang, Meimian Hua, Huan Chen, Bin Yang,

Ye Wang, **Qing Yang***


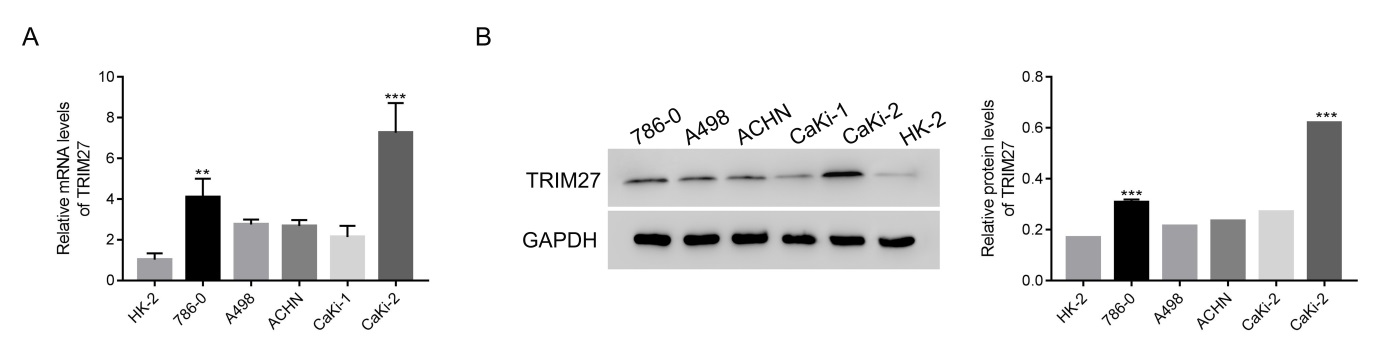


**Figure S1: TRIM27 was upregulated in human renal cancer cells.** A. The relative mRNA levels of TRIM27 were much higher in human renal 786-0 and Caki-2 cells than that of HK-2 cells. ** *p* < 0.01 vs HK-2, *** *p* < 0.001 vs HK-2. B. Western blot was performed to examine the protein contents of TRIM27 in HK-2, 786-0, A498, ACHN, CaKi-1 and CaKi-2 cells respectively. *** *p* < 0.001 vs HK-2.


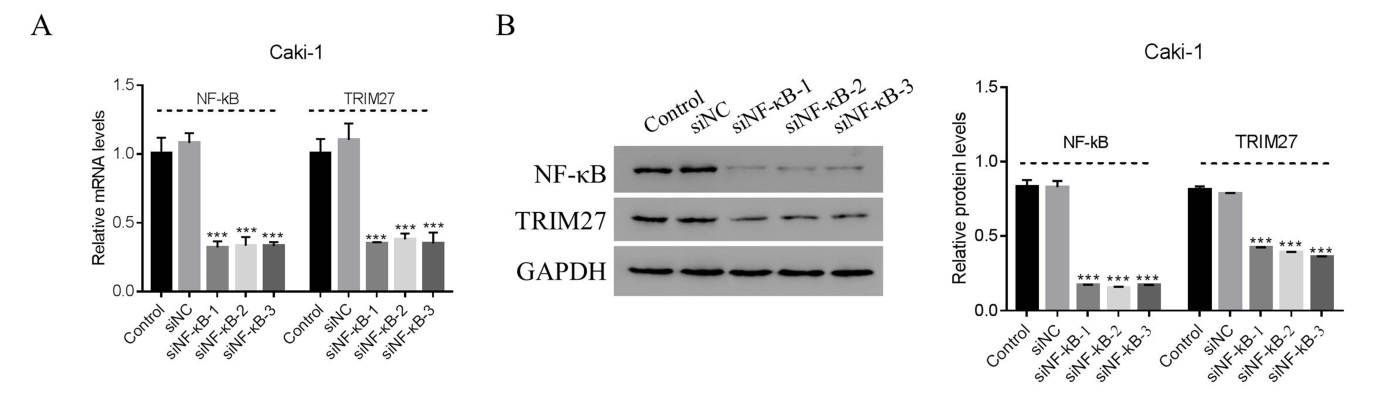


**Figure S2: siNF-κB inhibited the expression of TRIM27 in Caki-1 cells.** A and B. The relative mRNA and protein levels of NF-κB and TRIM27 in Caki-1 cells that transfecting with siNC, siNF-κB-1, siNF-κB-2 and siNF-κB-3 respectively. *** *p* < 0.001 vs siNC.
